# Coordination Mechanism and Bio-Evidence: Reactive γ-Ketoenal Intermediated Hepatotoxicity of Psoralen and Isopsoralen Based on Computer Approach and Bioassay

**DOI:** 10.3390/molecules22091451

**Published:** 2017-09-04

**Authors:** Yue Hai, Shan Feng, Lili Wang, Yetao Ma, Yiran Zhai, Zijun Wu, Sichao Zhang, Xin He

**Affiliations:** 1 School of Chinese Materia Medica, Tianjin University of Traditional Chinese Medicine, Nankai District, Tianjin 300193, China; haiyue09627@163.com (Y.H.); fsljever123@163.com (S.F.); wll1980@163.com (L.W.); Yetaoma@163.com (Y.M.); zhaiyr123@163.com (Y.Z.); lhd_zjw@163.com (Z.W.); 15202206506@163.com (S.Z.); 2 College of Pharmaceutical Sciences and Chinese Medicine, Southwest University, Chongqing 400715, China; 3 Tianjin State Key Laboratory of Modern Chinese Medicine, Tianjin 300193, China

**Keywords:** CYP3A4, γ-ketoenal intermediate, furanoepoxide, molecular docking, coordination compound, GSH depletion, hepatoxicity

## Abstract

Psoralen and isopsoralen are secondary plant metabolites found in many fruits, vegetables, and medicinal herbs. Psoralen-containing plants (*Psoralea corylifolia* L.) have been reported to cause hepatotoxicity. Herein, we found that psoralen and isopsoralen were oxidized by CYP450s to reactive furanoepoxide or γ-ketoenal intermediates, causing a mechanism-based inhibition of CYP3A4. Furthermore, in GSH-depleted mice, the hepatotoxicity of these reactive metabolites has been demonstrated by pre-treatment with a well-known GSH synthesis inhibitor, L-buthionine-S, Rsulfoxinine (BSO). Moreover, a molecular docking simulation of the present study was undertaken to understand the coordination reaction that plays a significant role in the combination of unstable intermediates and CYP3A4. These results suggested that psoralen and isopsoralen are modest hepatotoxic agents, as their reactive metabolites could be deactivated by H_2_O and GSH in the liver, which partly contributes to the ingestion of psoralen-containing fruits and vegetables being safe.

## 1. Introduction

Psoralen and isopsoralen are secondary plant metabolites found in many fruits (such as *Ficus carica* L.), vegetables (such as *Celery*), and medicinal herbs (such as *Psoralea corylifolia* L.). In addition, they are primarily used clinically to treat various skin diseases, such as psoriasis and vitiligo [1], since the US Food and Drug Administration approved the use of oral psoralens plus ultraviolet light A radiation (PUVA) to treat psoriasis in 1982. Modern pharmacological studies have further found that they are potential antimicrobial, antitumor, tyrosinase-activating, antidepressant, and estrogenic agents [2,3,4,5].

However, the ingestion of psoralens-containing plants, including the medicinal use of these compounds, can cause human health hazards. Bai-shi Wan and Zhuang-gu-guan-jie Wan are both proprietary Chinese medicines with *Psoralea corylifolia* (bu-gu-zhi) being the main constituent. There are 11 cases of liver injury associated with the use of Bai-shi Wan for the treatment of vitiligo in the Chinese literature from 1978 to 2005, and 47 cases of liver injury associated with the use of Zhuang-gu-guan-jie Wan for the treatment of degenerative osteoarthrosis in the Chinese literature from 2001 to 2008 [6,7]. Additionally, the Roussel Uclaf Causality Assessment Method (RUCAM) has been widely used to evaluate the causality of drug-induced liver injury (DILI) formation, and it can evaluate the liver damage caused by herbs and drugs [8,9]. Some causality assessments have been established to show a probable causality for extracts from psoralea leaves, which indicated the possibility that psoralen and isopsoralen reduce hapatotoxicity [10].

Since the 1980s, psoralen has been associated with hepatitis due to PUVA in humans [11,12]. Recently, animal studies have shown that psoralen and isopsoralen exhibit hepatotoxicity in mice, giving further evidence for psoralen and isopsoralen being hepatoxic agents in Bu-gu-zhi [12,13]. As ingredients that have widespread use in vegetables and Chinese medicines, it is important to understand the potential mechanism of psoralen- and isopsoralen-induced hepatotoxicity, and the consequential risks and benefits.

It is generally accepted that many chemicals require metabolic activation before they exert their toxic effects, e.g., methimazole, ticlopidine, and tienilic acid. Furan ring structure is one of the active functional groups that may produce reactive metabolites via CYP450s. The oxidation of the double bond of the furan ring to give the reactive furanoepoxide or γ-ketoenal intermediate, resulting in the modification of CYP450s, has been well-documented during the irreversible inactivation (also called mechanism-based inhibition) of CYP450s by the psoralen analogues 8-Methoxypsoralen (8-MOP), 5-Methoxypsoralen (5-MOP), and bergamottin [14,15,16,17]. Recently, psoralen and isopsoralen have also been found to give the irreversible inactivation of CYP1A2 in human and rat liver microsomes [18], and the formation of reactive metabolites has been reported [19,20]. Besides, some reactive furanoepoxide has been reported to be associated with hepatotoxicity or even carcinogenicity, such as the reactive epoxide of furan neoclerodane diterpenoids teucrin A, which can react with proteins leading to a mitochondrial permeability transition in rat and mouse hepatocytes [21], and aflatoxin B1-2,3-epoxide, which may covalently bind to DNA resulting in tumors [22]. Therefore, it is reasonable to presume that the formation of reactive metabolites is a plausible cause for psoralen and isopsoralen hepatotoxicity.

The molecular docking simulation approach has been widely employed towards understanding and predicting the possibilities of ligand–CYP450s interactions, especially in the discovery of candidate compounds, the exploration of potential targets, and the research of interaction mechanisms [23,24,25,26], but few papers have focused on studying and modeling the binding mode of mechanism-based inhibition with molecular docking so far.

**Scheme 1:**
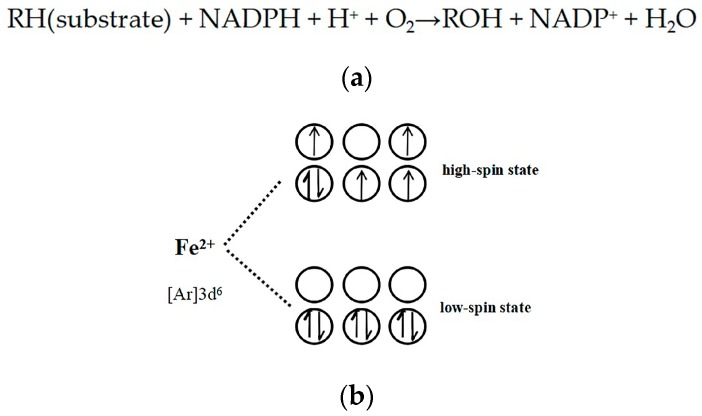
(**a**) The molecular mechanism of the CYP450s enzyme catalytic reaction; (**b**) Two spin states of Fe(II).

A large number of studies have shown that the activity center of CYP450s is iron porphyrin binding with a sulfur amino acid residue of half oboro glycine within a distance of 2.2 Å as a fifth axial coordination ligand [27,28]. Besides, chemical bonding and the shapes of molecular structures is a rather recent issue, and is explained by the concepts of hybrid orbitals, valence bond theory, Valence Shell Electron Pair Repulsion (VSEPR), resonance structures, and the outlet rule [29,30,31]. Meanwhile, there exists two spin states of Fe(II) influenced by the occupied ligand [32,33,34]. In the molecular mechanism of the CYP450s enzyme catalytic reaction (Scheme 1a), Fe^2+^ is more likely to be low-spin under the action of the ligand field, and the redox potential value of (Fe^2+^/Fe^3+^) is a minus, which contributes to Fe(II) being oxidized to Fe(III) (Scheme 1b).

To investigate this presumption, a mechanism-based inhibition (MBI) evaluation and in vitro liver microsomal incubation experiments were designed to determine and identify the reactive metabolites of psoralen and isopsoralen. A molecular docking simulation was applied to confirm the binding interaction of these unstable reactive metabolites on CYP3A4. The glutathione (GSH)-depleted agent L-buthi-onine-S, R-sulfoxinine (BSO) and a non-specific inhibitor of CYP450s, 1-Aminobenzotriazole (ABT), were administered to mice to elucidate the factors involved in psoralen- and isopsoralen-induced hepatotoxicity. Based on the results of the above studies, the mechanisms of psoralen- and isopsoralen-induced hepatotoxicity via reactive metabolites have been raised in the present study.

## 2. Results

### 2.1. MBI Evaluation of Psoralen and Isopsoralen

**Figure 1:**
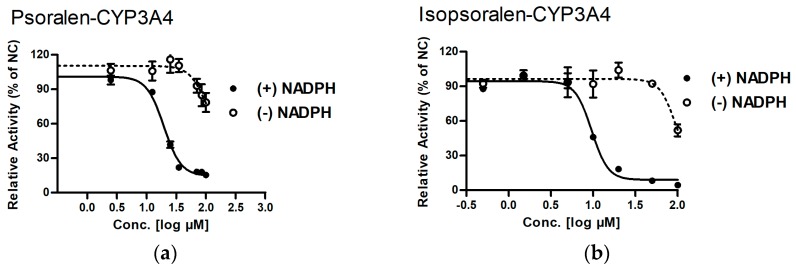

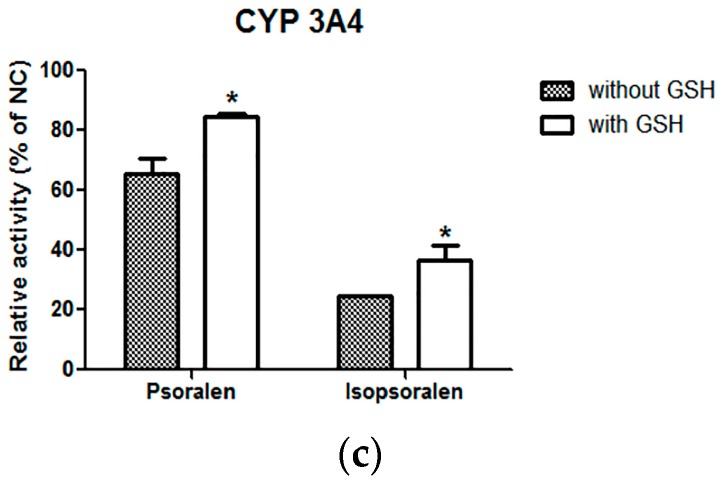
(**a**) *IC*_50_ shift of psoralen and isopsoralen for the different CYP450s in human liver microsomes (HLM) with (+) or without (–) NADPH pre-incubation; (**b**) *IC*_50_ shift of psoralen and isopsoralen for the different CYP450s in HLM with (+) or without (–) NADPH pre-incubation; (**c**) GSH was applied to interfere in the inhibition ability of psoralen and isopsralen (at *IC*_50_ concentration as detected in the *IC_50_ shift assay*) on CYP450s. Grey stylolitic shows the relative activity of CYP450s without adding GSH, while white stylolitic shows the restore activity of CYP450s by adding GSH. Results are presented as the mean ± SD from three independent experiments. * *p* < 0.05, significantly different from the corresponding incubations without cofactor GSH. NC is normal control.

MBI is a character for xenobiotics causing CYP450s inhibition via reactive metabolites, and the “*IC_50_ shift assay*” is a classic experiment for the determination of MBI. It has been reported that *IC*_50_ shift values at a magnitude of 1.2-fold to 3-fold indicate a positive finding [35]. In the present study, a 3-fold shift value was considered to be a positive finding. As shown in Table 1 and Figure 1a,b, the *IC*_50_ value of psoralen and isopsoralen on CYP3A4 was significantly decreased by the addition of NADPH during pre-incubation (*p* < 0.05, with shift values >3-fold). These data suggested that both psoralen and isopsoralen may be converted by CYP3A4 to highly reactive metabolites that bind to a catalytic site of these enzymes, causing irreversible enzyme inhibition. Scavenging agents (GSH) ameliorate the inhibition ability of psoralen and isopsralen. Most reactive metabolites are electrophilic in nature and can react with nucleophiles, such as GSH [36], hence the amelioration effects of GSH on the test compound’s inhibition ability for CYP450s may be evidence for reactive metabolites’ existence. In the present study, the addition of GSH (5 mM) ameliorated the inhibition ability of psoralen on CYP3A4 by 19.9% (*p* < 0.05, Figure 1c). The same occurred for isopsoralen: GSH ameliorated its inhibition ability on CYP3A4 by 12.3% (*p* < 0.05, Figure 1c). In general, reactive metabolites are scavenging agents for GSH with the ability to ameliorate the inhibition ability of psoralen and isopsralen on CYP450s via the trapping portion of their reactive metabolite.

### 2.2. Metabolism of Psoralen and Isopsoralen in Liver Microsomes

**Figure 2:**
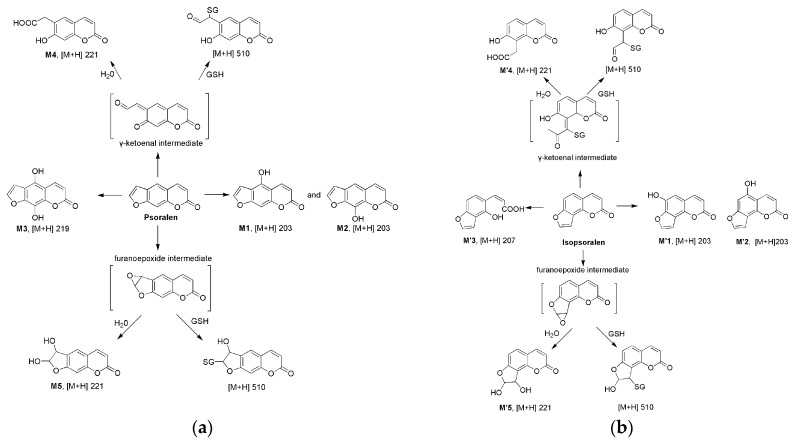
Proposed metabolic pathways of psoralen (**a**) and isopsoralen (**b**) in HLM and MLM: hydroxylation metabolites, hydrolysis metabolites, and furan ring oxidation metabolites (the most important metabolic pathway). The structures shown in brackets are the postulated reactive metabolites of psoralen and isopsoralen; electrophilic furanepoxides and the γ-ketoenal intermediate can also be deactivated with H_2_O or GSH.

Psoralen. Five new components were observed in psoralen metabolism samples following incubation with human liver microsomes (HLM) or mice liver microsomes (MLM) (Figure 2, Table 2). The formula, retention time, prominent fragment ions (MS2 spectrum in positive mode), and peak area (MRM mode) information of these metabolites is summarized in Table 2. The common characteristic of the five metabolites is the loss of small neutral molecules from precursor ions, such as a H_2_O (18 Da), CO (28 Da), and CO_2_ (44 Da). In addition, the [M + H]^+^ ion of M1, M2, and M3 respectively exhibited a *m*/*z* 16 Da, 16 Da, and 32 Da mass shift from psoralen, indicating that M1, M2, and M3 are hydroxylation metabolites (Table 2). The [M + H]^+^ ion of M4 and M5 is 221, which suggests that they are hydrolysis metabolites of the reactive furanoepoxide orγ-ketoenal intermediate (which will be discussed later). As the peak area (resulting from MRM mode) reflected (Table 2), both M4 and M5 are the most important metabolites of psoralen, suggesting that the formation of reactive metabolites was the main pathway for psoralen metabolism in HLM and MLM.

Isopsoralen. As the isomeride of psoralen, the metabolic pathway of isopsoralen in HLM and MLM was similar to that in psoralen (Figure 2, Table 2). The difference is that the [M + H]^+^ ion of 219 has not been detected, while the [M + H]^+^ ion of 205 has been detected in liver microsome incubations. The [M + H]^+^ ion of 205 (M′3) exhibits a *m*/*z* 18 Da mass shift from isopsoralen, indicating that M′3 is the hydrolysis metabolite of isopsoralen.

These data suggest that both psoralen and isopsoralen have a similar metabolic pathway in HLM and MLM, so it is then reasonable for the application of mice in the next reactive metabolite-induced hepatotoxicity study.

### 2.3. Identifying Reactive Metabolites Based on Mechanism-Based Inhibition

The reactive metabolite of psoralen and isopsoralen is presumed to have a molecular weight of 202 and cannot stably exist in nature. However, it is possible to deactivate with water ([M + H]^+^ = 221), forming a dihydrodiol metabolite or a 6-carboxymethyl-7-hydroxyl-coumarin metabolite; or deactivate with reduced GSH ([M + H]^+^ = 510), resulting in the formation of a GSH-reactive metabolite adduct. The observation of these metabolites in liver microsomal samples indicates the existence of a reactive metabolite.

**Figure 3:**
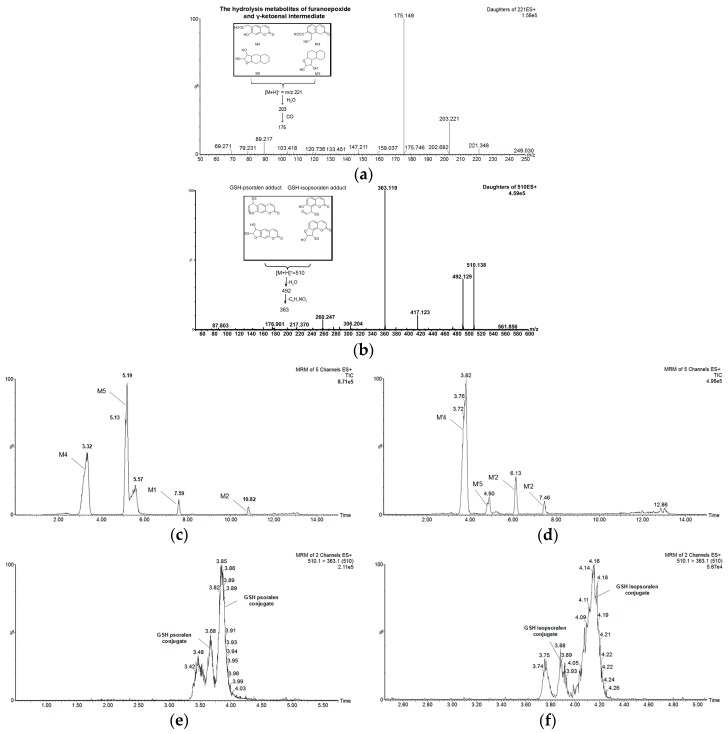
The fragmentation information for the psoralen and isopsoralen reactive metabolites formed in HLM and MLM. (**a**) The hydrolysis metabolites of furanoepoxide and the γ-ketoenal intermediate gave the protonated molecule [M + H]^+^ at *m*/*z* 221, and the MS2 spectrum showed prominent ions at *m*/*z* 203 [M + H − H_2_O]^+^ and 175 [M + H − H_2_O − CO]^+^; (**b**) the GSH-reactive metabolite adduct gave the protonated molecule [M + H]+ at *m*/*z* 510, and the MS2 spectrum showed prominent ions at *m*/*z* 492 [M + H − H_2_O]^+^ and 363 [M + H − H_2_O − 129]^+^; (**c**) (for psoralen) and (**d**) (for isopsoralen), multiple reaction monitoring chromatogram (*m*/*z* 221→175) of the hydrolysis metabolites of reactive metabolites; (**e**) (for psoralen) and (**f**) (for isopsoralen), multiple reaction monitoring chromatogram (*m*/*z* 510→393) GSH-reactive metabolites.

Identification of 6-carboxymethyl-7-hydroxyl-coumarin and Dihydrodiol metabolites. Two metabolites with [M + H]^+^ at *m*/*z* 221 are observed, respectively, in psoralen (M4 and M5) and isopsoralen (M′4 and M′5) metabolism incubations (Table 2). All of the hydrolysis-reactive metabolites share the same fragmentation information (MS2 spectrum in positive mode) as shown in Figure 3a. Based on structurally specific fragmentation, an MRM method has been developed and all the hydrolysis-reactive metabolites have been detected (Figure 3c,d) and quantified (Table 2), suggesting that they are the main metabolites of psoralen or isopsoralen. However, due to 6-carboxymethyl-7-hydroxyl-coumarin containing carboxy, it can be detected in negative-scanning mode, while only M4 (or M′4) can be detected in negative-scanning mode with the [M − H]^−^ ion at 219.

These data suggest that M4 or M′4 (containing carboxy) are 6-carboxymethyl-7-hydroxyl-coumarin, the hydrolysis metabolite of the γ-ketoenal intermediate, while M5 or M′5 are dihydrodiol metabolites, the hydrolysis metabolite of furanoepoxide.

GSH trapping of reactive metabolite. In the human and mice liver incubations, after the addition of GSH, two GSH conjugates of the reactive metabolites with [M + H]^+^ = 510 were respectively found for psoralen and isopsoralen (for psoralen, the retention time (R_t_) was 3.68 min and 3.86 min; for isopsoralen, the *R*_t_ was 3.88 and 4.15 min). These data suggested that both the γ-ketoenal intermediate and furanoepoxide can be trapped by GSH to form a GSH conjugate. All of the GSH conjugates of the reactive metabolites share the same fragmentation information (MS2 spectrum in positive mode) as shown in Figure 3b. These GSH conjugates can be detected with the same MRM method (Figure 3e,f). In fact, the GSH conjugate of the reactive metabolites cannot be distinguished in the results from the γ-ketoenal intermediate or furanoepoxide in our experimental conditions.

These data suggested that both psoralen and isopsoralen could generate the formation of reactive furanoepoxide and γ-ketoenal intermediate, followed by a GSH attack to form GSH adducts, or a hydrolytic attack to form hydrolysis metabolites, in both HLM and MLM.

### 2.4. Evidence for Reactive Metabolites-Induced Hepatotoxicity

Reduced GSH represents the most important biomolecule that protects cells against chemical-induced cytotoxicity [37]. GSH-depleted animal models are a standard, commonly used model to investigate the underlying mechanisms of hepatotoxicity caused by the reactive metabolites of xenobiotic compounds [37,38,39,40]. Additionally, then, the present study applied BSO, a well-known GSH synthesis inhibitor, and constructed a GSH-depleted mice model to evaluate the hepatotoxicity induced by psoralen and isopsoralen reactive metabolites.

**Figure 4:**
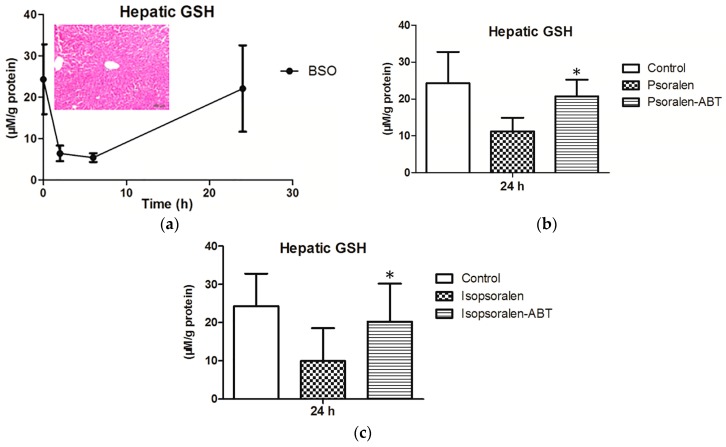
(**a**) The hepatic GSH level curve after treatment with BSO alone in 24 h, and the inserted pathological section suggested that BSO was without hazard to the liver during the period; (**b**) and (**c**), the hepatic GSH level after treatment with psoralen (or isopsoralen) in the absence (or presence) of ABT at 24 h (* *p* <0.05 compared with test compound group).

Assessment of psoralen hepatotoxicity. After treatment with BSO, the hepatic GSH level displayed a time-dependent decrease at least for 6 h, while returning to a normal level at 24 h and without hazard to the liver during the period (Figure 4a), which is in accordance with previous reported GSH-depleted mice models [38]. After treatment with psoralen, the GSH level decreased to 11.26 ± 3.67 µM/g protein at 24 h, while ABT prevented the decrease (*p* < 0.05, Figure 4b).

**Figure 5:**
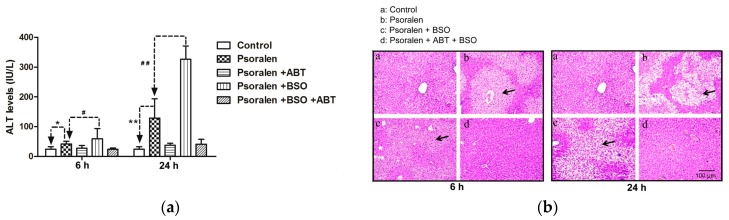
(**a**) The histogram shows the time course of ALT levels (IU/L), while the pathological section (**b**) shows the histopathological evaluation of mice after administration of vehicle (control), oral psoralen (300 mg/kg) alone, or a combination of BSO (650 mg/kg) and intraperitoneal ABT (100 mg/kg). Black arrows indicate pathological changes. * *p* < 0.05, ** *p* < 0.01 in comparison with control group. ^#^ *p* < 0.05, ^##^ *p* < 0.01 in comparison with psoralen group.

Figure 5 shows the time course of mice plasma ALT activities after the administration of psoralen alone or in combination with BSO. Sole treatment with psoralen caused only a marginal increase (6 h: 1.8-fold over the controls, 24 h: 5.3-fold over the controls) in serum ALT activity. In combination with BSO, psoralen treatment resulted in a significant time-dependent increase (6 h: 2.4-fold over the controls, 24 h: 14-fold over the controls) in serum ALT activity. Additional treatment with the CYP450s inhibitor ABT completely suppressed the elevation of ALT activity caused by the treatment of psoralen alone or with BSO (Figure 5). Sole treatment with either BSO or ABT produced no significant changes in plasma ALT activity at 24 h (Figure 5).

A histopathological evaluation showed diffuse granular degeneration at 6 h after psoralen treatment, and diffuse vacuolation was observed at 24 h (Figure 5b). Co-treatment with BSO did not aggravate the histopathology at 6 or 24 h (Figure 5b). Additionally, ABT completely relieved the granular degeneration and vacuolation of the centrilobular hepatocytes that was caused by the treatment of psoralen alone or in combination with BSO (Figure 5b).

**Figure 6:**
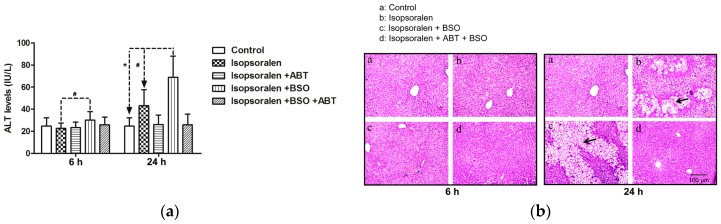
(**a**) The above histogram shows the time course of ALT levels (IU/L), while the pathological section (**b**) shows the histopathological evaluation of mice after administration of vehicle (control), oral isopsoralen (300 mg/kg) alone, or a combination of BSO (650 mg/kg) and intraperitoneal ABT (100 mg/kg). Black arrows indicate pathological changes. ** *p* < 0.01 in comparison with control group. ^#^ *p* < 0.05 in comparison with isopsoralen group.

Assessment of isopsoralen hepatotoxicity. After treatment with isopsoralen, the GSH level decreased to 9.94 ± 8.57 µM/g protein at 24 h, while ABT prevented the decrease (*p* < 0.05, Figure 4c). Treatment with isopsoralen alone did not affect the serum’s ALT activity at 6 h (22.73 IU/L, Figure 6a), while a slight increase in the ALT activity was observed at 24 h (43.20 IU/L, Figure 6a). The plasma ALT activity in mice treated with isopsoralen in combination with BSO was slightly increased (22.73 to 30.08 IU/L at 6 h, *p* > 0.05; 43.20 to 68.99 IU/L at 24 h, *p* > 0.05). ABT completely suppressed the elevation of ALT activity caused by isopsoralen alone or in combination with BSO at 24 h (Figure 6a).

A histopathological evaluation revealed weak granular degeneration at 6 h Figure 6b), and diffuse vacuolation was observed at 24 h after treatment with isopsoralen alone (Figure 6b). Co-treatment with BSO did not aggravate the histopathology at 6 or 24 h (Figure 6b). Additionally, ABT completely relieved the granular degeneration and vacuolation of the centrilobular hepatocytes that was caused by the treatment of isopsoralen alone or in combination with BSO (Figure 6b).

### 2.5. Evidence for the Reactive γ-Ketoenal Metabolite–CYP3A4 Heme Fe(II) Coordination Complex

**Figure 7:**
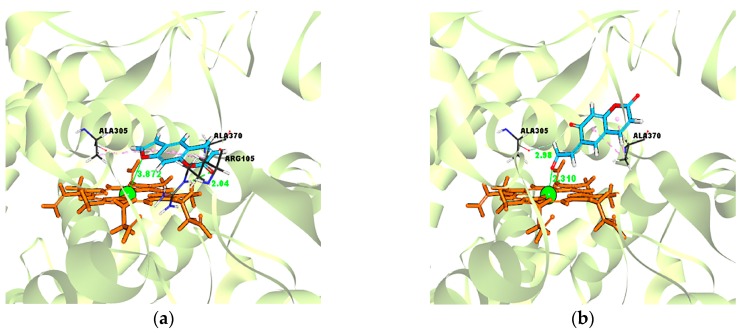

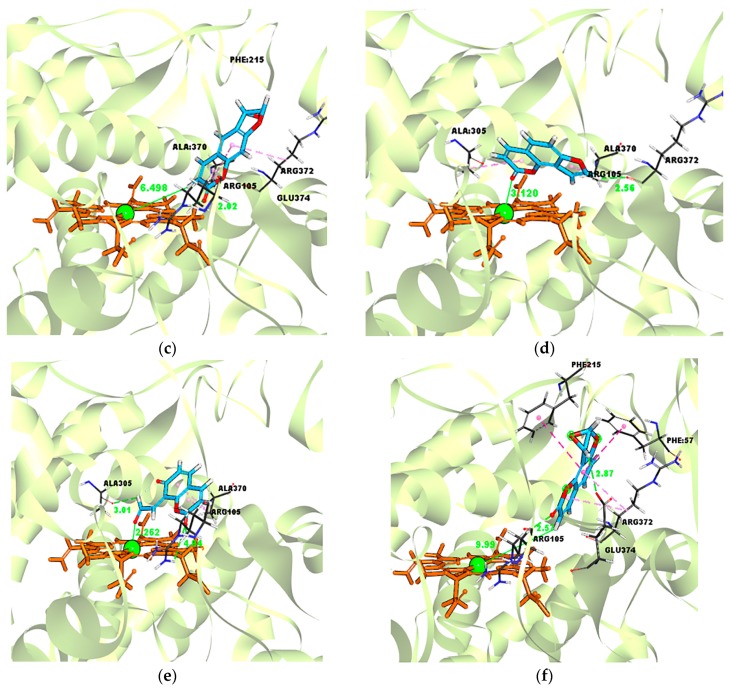
Docking simulation of Ligand into CYP3A4. (**a**) Psoralen–CYP3A4; (**b**) γ-ketoenal intermediate of psoralen–CYP3A4; (**c**), Furanoepoxide intermediate of psoralen–CYP3A4; (**d**) Isopsoralen–CY3A4; (**e**) γ-ketoenal intermediate of isopsoralen–CYP3A4; (**f**) Furanoepoxide intermediates of isopsoralen–CYP3A4. The heme and iron atoms are colored with brown and green, respectively.

A molecular docking simulation was performed to provide deep insight into the binding modes of the ligand–CYP3A4 enzyme complex. As shown in Figure 7 and Table 3, psoralen and its metabolism intermediates (γ-ketoenal and furanoepoxide) bound to the same active cavity of human CYP3A4 with a −Cdocker energy of 19.4697 kcal/mol, 21.4426 kcal/mol, and −21.3421 kcal/mol, respectively. On the other hand, isopsoralen and its metabolism intermediates (γ-ketoenal and furanoepoxide) bound to the same active cavity of human CYP3A4 as well, with a –Cdocker energy of 18.5824 kcal/mol, 19.635 kcal/mol, and −23.4627 kcal/mol, respectively. Notably, Figure 7a,d shows that psoralen and isopsoralen could bind the Arg 105 amino acid residue via a hydrogen binding interaction within a distance of about 2.04 Å and 2.56 Å, which illustrates that the H-bond interactions play an important role in the binding. In addition, the Ligand–Central ion distance of psoralen–CYP3A4, the γ-ketoenal intermediates of psoralen–CYP3A4, and the furanoepoxide intermediate of psoralen–CYP3A4 was 3.872 Å, 2.310 Å, and 6.498 Å, respectively (Figure 7a–c). Meanwhile, the Ligand–Central ion distance of isopsoralen–CYP3A4, the γ-ketoenal intermediate of isopsoralen–CYP3A4, the and furanoepoxide intermediate of isopsoralen–CYP3A4 was 3.120 Å, 2.226 Å, and 9.993 Å, respectively (Figure 7d–f).

These results suggest that Arg 105 was a key amino acid residue for the binding of psoralen/isopsoralen with CYP3A4 via hydrogen binding interactions. Besides, the γ-ketoenal intermediates of psoralen/isopsoralen could adapt themselves better in the active site, with a Fe–ligand distance that was about 2.31 Å and Å less than the others and a functional score that was about 21.4426 kcal/mol and 19.635 kcal/mol higher than the others, which implies that the coordination mechanism could result in mechanism-based inhibition 2.226.

## 3. Discussion

In the present study, five metabolites of psoralen and isopsoralen were found in HLM and MLM: (1) hydroxylation metabolites; (2) hydrolysis metabolites; and (3) furan ring oxidation metabolites (Figure 2), suggesting the similar metabolic pathway of 8-MOP [41]. As the peak area reflected (Table 1), both M4 and M5 (M′4 and M′5) are the main metabolites of psoralen (isopsoralen), suggesting that the formation of reactive metabolites was the main pathway for psoralen/isopsoralen metabolism in HLM and MLM. Subsequently, the trapping of furanoepoxide and γ-ketoenal intermediate by GSH provides further evidence for the existence of reactive metabolites, and their irreversible inactivation of CYP3A4 (Figure 1). Furthermore, as the *IC*_50_ shift folds reflected (Table 1), psoralen causes a more significant *IC*_50_ shift than isopsoralen, suggesting that the reactive metabolite of psoralen has a more potent electrophilicity to covalently bind to proteins.

Due to the low electron density, reactive metabolites can react with cellular components, such as proteins, DNA, and membranes (e.g., mitochondrial membrane), resulting in cell stress, which is involved in the occurrence of liver toxicities [42,43,44]. In a study of 207 of the most commonly prescribed oral medications in the U.S., 62–69% of compounds with reactive metabolite formation were associated with drug-induced liver injury [44]. GSH-depleted animal models are a standard, commonly used model to investigate the underlying mechanisms of hepatotoxicity caused by reactive metabolites [40]. In the present study, BSO significantly reinforced the psoralen (300 mg/kg) hepatotoxicity in mice by increasing the plasma ALT activities (6 h: from 42.17 to 59.90 IU/L; 24 h: from 128.42 to 326.07 IU/L). Treatment with ABT, a non-specific inhibitor of CYP450, resulted in a remarkable protection against the hepatotoxicity of psoralen or isopsoralen alone or in combination with BSO (Figure 5 and Figure 6). Overall, these results suggest that the reactive metabolites of psoralen and isopsoralen are responsible for their hepatotoxicity. Furthermore, the hepatotoxicity of isopsoralen is weaker than that of psoralen, which can be explained by the fact that isopsoralen metabolism results in reactive metabolites with less electrophilicity than that of psoralen (Table 1). Through a molecular docking analysis, the -Cdocker energy score value of psoralen γ-ketoenal intermediates was found to be slightly higher than that of isopsoralen, which is consistent with the *IC_50_* *shift assay* result that psoralen metabolism produces reactive metabolites with higher mechanism-based inhibitory effects than that of isopsoralen (Table 3).

**Figure 8:**
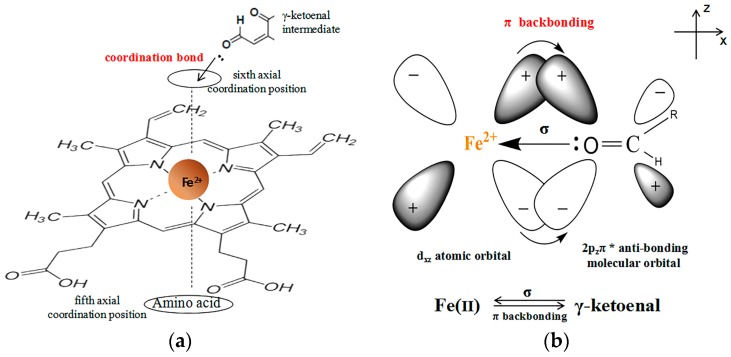
(**a**) The illustration of the γ-ketoenal intermediate of the psoralen/isopsoralen–heme Fe(II) CYP3A4 coordination compound; (**b**) The illustration of the σ and π backbonding interaction between γ-ketoenal intermediates and heme Fe(II) of CYP3A4: the mechanism of coordination binding between γ-ketoenal intermediates and heme Fe(II) of CYP3A4.

In the present study, psoralen and isopsoralen were oxidized by CYP450s, causing the mechanism-based inhibition (MBI) of CYP3A4. Although the mechanism of MBI is unknown for now, we can find some clues from the molecular docking study. On one hand, the psoralen and isopsoralen γ-ketoenal intermediates bound to human CYP3A4 more tightly (21.4426 kcal/mol, 19.635 kcal/mol, respectively) than psoralen/isopsoralen (19.4697 kcal/mol, 18.5824 kcal/mol, respectively) and the furanoepoxide intermediates (−21.3421 kcal/mol, −23.4627 kcal/mol). On the other hand, according to the bond valence model [45,46,47] and the empirical data of published bond lengths from the Inorganic Crystal Structure Database (ICSD) and the Cambridge Structure Database (CSD) [45,48], the O atom of the γ-ketoenal intermediate of psoralen/isopsoralen, which can provide one lone electron pair, is close to the iron capable of accepting a lone electron pair on the sixth axial coordination position with a distance of 2.31 Å and 2.226 Å, suggesting that the γ-ketoenal intermediates of psoralen/isopsoralen have the possibility to coordinate to bond to CYP3A4 (Figure 8a). In this occasion, electrons will be concentrated around the central ion, which is not in accordance with the electric neutrality principle [49]. Therefore, we have reason to believe that there exists a π backbinding [50,51], in which electrons are transferred from the d_xz_ atomic orbital of central ion Fe^2+^ to the 2p_z_π* anti-bonding molecular orbital on the O=C group of the γ-ketoenal intermediates’ ligand. The correlation of this coordination binding can be described as Figure 8b. These findings may account for the mechanism of coordination binding as a vital role in mechanism-based inhibition between the γ-ketoenal intermediates and CYP3A4. Consistently, high-dose 5-MOP (560 mg/kg daily for 12 days) has been shown to cause hepatocyte changes in animals [52]. In humans, transient elevations of liver enzymes were found in 5% of patients (4 of 80) within 3.5–20 months of commencement of PUVA 5-MOP [53]. However, there was no liver injury report associated with 8-MOP till now. In the present and previous study, psoralen, isopsoralen, and 5-MOP were found to form furanoepoxide or γ-ketoenal intermediate via CYP450s, while 8-MOP was only found to form furanoepoxide [17,18]. It is then presumed that γ-ketoenal intermediate may be the more plausible cause for the hepatotoxicity of psoralen, isopsoralen, and 5-MOP; however, further studies need to confirm this.

Apiaceous vegetables (which includes carrots, celery, parsnips, parsley, dill, cilantro, etc.) are rich in furanocoumarins psoralen, 5-MOP, and 8-MOP [54], but there has been no hepatotoxicity report associated with these vegetables to present. In contrast, Bu-gu-zhi (the recommended dose is 6–15 g/day, with psoralen and isopsoralen as the main ingredients) has four case reports of liver injury, and one case of acute cholestatic hepatitis associated with the use of Bu-gu-zhi over 10 times the usual dosage [52,53]. To present, there are no data on the therapeutic or toxic dosage of psoralen in humans. In animal studies of oral psoralen or isopsoralen 40 mg daily for 28 days in mice, liver injury by psoralen or isopsoralen was associated with elevated serum ALT, AST, and/or ALP levels [55,56]. As a mechanism exploration study, a psoralen and isopsoralen dose of 300 mg/kg was set at approximately one-half the oral LD50 value (615 mg/kg in mice), and liver injury was observed as mild pathology damage (granular degeneration) after a single oral psoralen and isopsoralen dose. Together, all the studies suggest that both psoralen and isopsoralen are modest hepatotoxic agents, which can be partly explained by the fact that furanoepoxide or the γ-ketoenal intermediate can be deactivated with H_2_O and GSH in liver in the present study. This also provides support for the safe ingestion of apiaceous vegetables, while other factors (such as psoralens content in vegetables) should also be considered. In addition, the co-administration of GSH could be considered while taking psoralens or psoralen-containing plants in order to avoid or relive liver injury in a clinical setting. The present study identified the reactive furanoepoxide or γ-ketoenal intermediate produced by the CYP450-mediated metabolism of psoralen and isopsoralen, and then how it binds to CYPs or other macromolecules. Thus, we believe that psoralen and isopsoralen may cause idiosyncratic drug-induced liver injury, which is responsible for hepatotoxicity in mice.

The ethanol extracts of the seeds of Bu-gu-zhi have been proposed as a food additive for the preservation of some processed foods in Japan. Takizawa et al found that long-term (90 days) ingestion of the extracts of the seeds of Bu-gu-zhi showed testicular toxicity in rats [56]. Interestingly, CYP2D4/18 mRNAs are expressed in rat testis [57], while the rat and human CYP2D isoforms share a high sequence identity (>70%) [58]. Then, it is presumed that the reactive metabolites are maybe a plausible cause for Bu-gu-zhi rat testicular toxicity, while the related toxicity in humans and its underlying mechanism need to be further considered.

According to clinical experience, the chemical composition and pharmacological activity of traditional Chinese medicine is very complicated, and it is very important to avoid long term treatment and inappropriate medication to prevent liver injury from Traditional Chinese Medicine (TCM), but there are few precise and effective ways to solve this problem. Thus, we believe RUCAM can play a bigger role in providing prediction for safe and rational drug use [8,9].

## 4. Materials and Methods

### 4.1. Inhibitory Effects of Psoralen and Isopsoralen on CYP3A4

Psoralen, isopsoralen, phenacetin, acetaminophen, and midazolam maleate were obtained from the National Institute for the Control of Pharmaceutical and Biological Products (Beijing, China). β-nicotinamide adenine dinucleotide phosphate (NADPH), dextromethorphan, dextrorphan, and 1-hydroxymidazolam were purchased from Sigma Chemical Co. (St. Louis, MO, USA). l -buthi-onine-*S*,*R*-sulfoxinine (BSO) was obtained from Acros Organics (New Jersey, NJ, USA), and 1-Aminobenzotriazole (ABT) was purchased from Tokyo Chemical Industry Co., LTD (Tokyo, Japan). All other reagents were of analytical or high-performance liquid chromatography grade. Pooled human liver microsomes (HLM), which were purchased from the Research Institute for Liver Diseases Co., Ltd. (Shanghai, China), were prepared from human liver tissues under Chinese organ donation regulations with the full consent of the patients. Mice liver microsomes (MLM) were purchased from the Research Institute for Liver Diseases Co., Ltd. (Shanghai, China). Liver microsomal samples were stored at −80 °C until further use.

### 4.2. MBI Evaluation of Psoralen and Isopsoralen

*IC_50_ shift assay*. In the present study, an *IC_50_ shift assay* was performed using a two-step procedure. First, psoralen and isopsoralen at seven concentrations (from 2.5 to 100 µM, except for a psoralen test of 200 µM on CYP3A4) were pre-incubated with HLM for 30 min at 37 °C with/without (+/−) NADPH. After pre-incubation, a 0.02 mL aliquot of the mixture was added to a mixture containing a standard CYP substrate (final concentrations at the Km) in 0.18 mL of potassium phosphate buffer to measure CYP activities. After incubation for 15 min at 37 °C, the reactions were ceased by adding four volumes of pre-cooled methanol. The supernatants were centrifuged at 14,000 rpm for 15 min and then stored at −20 °C until the LC/MS/MS analysis of metabolites.

### 4.3. Effects of Scavenging Agents (GSH) on Inhibition Ability of Psoralen and Isopsralen

In the present study, GSH was applied to interfere with the inhibition ability of psoralen and isopsralen (at *IC*_50_ concentration as detected in *IC_50_ shift assay*) on CYP3A4, and the incubation procedure was the same as for the “*IC_50_ shift assay* (with NADPH)” experiment, except for the addition of GSH (5 mM at final incubations).

Analysis of samples. The LC/MS/MS method was conducted as described in [59].

### 4.4. Metabolism of Psoralen and Isopsoralen in Liver Microsome

Phase I Metabolism. Psoralen or isopsoralen (50 μM) was suspended in 150 μL of buffer containing 0.1 M potassium phosphate (pH 7.4) and HLM (or MLM) (1.0 mg/mL) and was pre-incubated at 37 °C for 5 min. Subsequently, 150 μL of NADPH solution (final concentration of 1 mM) was added to each reaction mixture. After incubation at 37 °C for 90 min in a shaking water bath, the reaction was terminated by adding 600 μL of ice-cold methanol. The contents were vortexed for 30 s and then centrifuged at 4000 rpm for 15 min. The metabolites of psoralen and isopsoralen in the supernatant were identified by UPLC-MS/MS.

GSH capture of reactive metabolites. The incubation method was the same as for Phase I Metabolism, execpt for the addition of GSH (final concentration of 5 mM).

Metabolite analysis by UPLC-MS/MS. The UPLC/MS/MS method was carried out using a Waters Acquity UPLC Sample Manager and a Waters Acquity UPLC Binary Solvent Manager connected to a Waters Quattro Premier XE triple-quadruple mass spectrometer equipped with a combined ESI probe and Mass Lynx 1.4 software (Waters, Milford, MA, USA). An Acquity UPLC BEH C18 column (1.7 µm, 2.1 mm × 100 mm) was used. The instrument settings were as follows: ESI+; source temperature, 120 °C; dissolution temperature, 350 °C; capillary voltage, 3.2 kV; dissolution N_2_, 600 L/h; cone N_2_, 50 L/h. Acquisition was carried out from *m*/*z* 50 Da to 1000 Da in the total ion scan mode. MS/MS spectra were acquired from *m*/*z* 50 Da to 600 Da in the daughter ion scan mode. The solvent system consisted of solvent A (0.1% CH_3_COOH) and solvent B (100% ACN). The flow rate was 0.2 mL/min. The gradient elution method was 5 to 80% B over 12 min, followed by 5% B from 12 to 15 min.

Quantitation analysis of psoralen and isopsoralen metabolites by UPLC-MS/MS. The UPLC/MS/MS MRM method was developed to permit the detection and quantitation of psoralen (or isopsoralen) metabolites based on structurally specific fragmentation obtained from collision-induced dissociation: M1, M2, M′1, or M′2 *m*/*z* 203.3→147.1; M3 *m*/*z* 219.4→157.5; M′3 205→177.1; M4, M5, M′4, or M′5 *m*/*z* 221→174.9; GSH-reactive metabolite adducts *m*/*z* 510→363.1. The HPLC method and electrospray ionization were performed with the same conditions as those for metabolite analysis by UPLC-MS/MS in liver microsomes, except for the cone (30 V) and collision energy (20 V).

### 4.5. Animals, Treatment, and Sampling

For the mechanism exploration study, a psoralen and isopsoralen dose of 300 mg/kg was set at approximately one-half the oral LD50 value (615 mg/kg in mice) in the present study. Male 6-week-old KunMing (KM) mice were purchased from the Animal Institute of the Tianjin University of Traditional Chinese Medicine (Tianjin, China), and were used after a 1-week acclimatization period. The animals were housed in mice cages in a unidirectional airflow room under controlled conditions (20–24 °C, 12:12-h light:dark cycles, and free access to water). We assured that all animals received humane care according to the criteria outlined in the “Guide for the Care and Use of Laboratory Animals” prepared by the National Academy of Sciences and published by the National Institutes of Health ((NIH) publication 86–23 revised 1985).

In the hepatotoxicity study, the mice were randomly assigned to 22 groups (*n* = 10/group). The mice were intraperitoneally injected with BSO (650 mg/kg) in saline (0.2 mL/20 g). The intraperitoneal dosing of ABT (100 mg/kg) in saline (0.2 mL/20 g) was simultaneous with the BSO dosing. One hour later, the mice were orally administered psoralen and isopsoralen (300 mg/kg) in 0.5% carboxymethylcellulose sodium (0.2 mL/20 g). Food was removed 14–16 h before BSO dosing, and was supplied again 6 h after administration of the test compounds. At 6 and 24 h, blood was collected by eyeball removal, and then the liver was removed. Plasma was separated from these blood samples, and alanine aminotransferase (ALT) was determined with a commercial kit, the GPT-UV test (Nanjing jiancheng Bioengineering Institute). A piece of each liver sample was fixed in 10% formalin, embedded in paraffin wax, sectioned, and stained with hematoxylin and eosin for histopathological evaluation. For the groups treated with psoralen/isopsoralen in the absence/presence of ABT, or groups treated with BSO alone, the hepatic GSH has been determined.

### 4.6. Molecular Docking Simulation

**Scheme 2:**
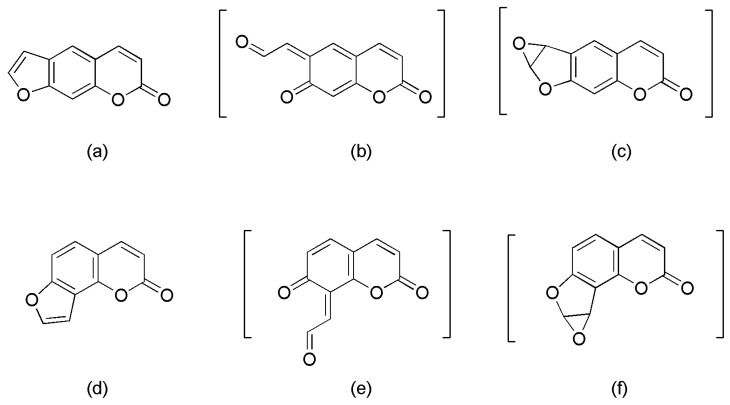
Chemical structures of docking ligands. (**a**) Psoralen; (**b**) γ-ketoenal intermediate of psoralen; (**c**) Furanoepoxide intermediate of psoralen; (**d**) Isopsoralen; (**e**) γ-ketoenal intermediate of isopsoralen; (**f**) Furanoepoxide intermediate of isopsoralen.

Ligand structure preparation. The structures of psoralen (ID: 6199) and isopsoralen (ID: 10,658) were downloaded from the PubChem database. Combining a recent report and our above study [20,21], the structure of furanoepoxide and γ-ketoenal intermediates were edited from *ChemDraw Ultra 16.0* (Scheme 2). All compounds were prepared and optimized with a CharMm force field by using the Ligand Preparation module of *Discovery Studio software 4.5*.

Protein Structure Preparation. Here, the X-ray crystallographic structures of the human CYP3A4 (Protein Data Bank code: 1W0F) were obtained from the Protein Data Bank. For the docking study, a protein preparation module was utilized to optimize the structure of CYP3A4 by protonating, removing water, building missing side chains, and then adding a forcefield CharMm.

Molecular Docking simulation. This process was carried out by Cdocker module, a grid-based molecular docking method that employs CharMm [60]. With Cdocker, the initial ligand conformations were sampled via high temperature molecular dynamics and were also allowed to flex during the refinement (via simulated annealing Moleculer Dynamic). Moreover, Cdocker also provided a physics-based scoring function via the CharMm energy of the docked complex. Cdocker has been shown to give highly accurate docked poses [61].

The conformations of small molecules were flexible, while protein atoms were fixed to their positions of *Discovery Studio 4.5*. Every ligand was put into an active site. To ensure the docked poses were diverse, we set the Pose Cluster Radius to 0.5 Å, and the rest of the parameters were set to their defaults. After varying the model, a 1000-step algorithm minimization was carried out to obtain a low-energy conformation without steric clashes in protein structure. The result was evaluated by an empirical function −Cdocker energy and iron–ligand distance was recorded.

### 4.7. Data Analysis

*IC*_50_ value calculation. The *IC*_50_ value was calculated using GraphPad Prism (version 4.0) software (GraphPad Software, San Diego, CA, USA).

Statistical analysis. The results from the serum ALT assay were analyzed by Kruskal–Wallis non-parametric analysis of variance followed by Mann–Whitney’s U test. For the data of the GSH assay and CYP450s relative activity, comparisons between the two groups were made by independent-samples *t* test. SPASS 11.5 was used for these statistical analyses. Differences were considered significant when *p* < 0.05 or *p* < 0.01.

## 5. Conclusions

The present study identified the reactive furanoepoxide or γ-ketoenal intermediate produced by the CYP450-mediated metabolism of psoralen and isopsoralen, which are responsible for hepatotoxicity in mice, and provided the new thought to study the mechanism of coordination binding between reactive metabolites and CYP450s using molecular docking.

## Figures and Tables

**Table 1:** *IC*_50_ of psoralen and isopsoralen on 3A4 with/without (+/−) NADPH pre-incubation.

*IC*_50_ (µM)				
Enzyme	Compound	(−) NADPH	(+) NADPH	Shift (fold)
CYP3A4	Psoralen	>200	19.50 ± 1.04 **	>10.3
	Isopsoralen	96.83 ± 2.21	9.59 ± 1.04 **	10.1

Each value is the mean ± SD of three experiments; ** *p* <0.01 compared with the *IC*_50_ value of the (−) NADPH condition.

**Table 2:** Metabolites of psoralen in HLM and mice liver microsomes (MLM), and their retention time, prominent fragment ions (MS2 spectrum), and peak area information.

	Formula	[M + H]^+^ *m*/*z*	Retention Time (min)	Prominent Fragment Ion (*m*/*z*)	Peak Area ^1^	
					HLM	MLM
Psoralen	C_11_H_6_O_3_	187	7.72	159, 143, 131, 115		
M1	C11H_6_O_4_	203	5.19	175.1, 147.0, 119.2	557	1019
M2	C_11_H_6_O_4_	203	7.58	175.2, 159.1, 147.1, 131.2	502	8655
M3	C_11_H_6_O_5_	219	7.23	201.1, 174.8, 157.5	529	164
M4	C_11_H_8_O_5_	221	3.34	203.1, 175.1, 158.9, 147.0	66,831	98,864
M5	C_11_H_8_O_5_	221	5.18	203.2, 175.1, 147.2	61,016	102,180
Isopsoralen	C_11_H_6_O_3_	187	8.06	158.6, 142.6, 130.6, 114.6		
M′1	C_11_H_6_O_4_	203	6.12	175.1, 147.0, 119.2	1288	14,513
M′2	C_11_H_6_O_4_	203	7.45	175.3, 159.0, 147.1, 131.2	1349	4707
M′3	C_11_H_10_O_4_	205	5.23	177.1,148.8, 133.0, 131.2	312	418
M′4	C_11_H_8_O_5_	221	3.80	203.3, 174.9, 159.4, 146.8	34,274	96,828
M′5	C_11_H_8_O_5_	221	5.01	203.3, 175.4	1627	7725

^1^, The UPLC/MS/MS MRM method was developed to permit the quantitation of psoralen (or isopsoralen) metabolites based on structurally specific fragmentation obtained from collision-induced dissociation: M1, M2, M′1, or M′2 *m*/*z* 203.3→147.1; M3 *m*/*z* 219.4→157.5; M′3 205→177.1; M4, M5, M′4, or M′5 *m*/*z* 221→174.9.

**Table 3:** Empirical function score value and iron–ligand distance of ligand–CYP3A4 by molecular docking simulation.

Ligand–Enzyme Complex	−Cdocker Energy	Ligand–Central Ion Distance
Psoralen–CYP3A4	19.4697 kcal/mol	3.872 Å
γ-ketoenal intermediate of psoralen–CYP3A4	21.4426 kcal/mol	2.310 Å
Furanoepoxide intermediate of psoralen–CYP3A4	−21.3421 kcal/mol	6.498 Å
Isopsoralen–CYP3A4	18.5824 kcal/mol	3.120 Å
γ-ketoenal intermediate of isopsoralen–CYP3A4	19.635 kcal/mol	2.226 Å
furanoepoxide intermediate of isopsoralen–CYP3A4	−23.4627 kcal/mol	9.993 Å
